# 4-[4-Eth­oxy­carbonyl-5-(3,4-methyl­ene­dioxy­phen­yl)-3-oxocyclo­hex-1-en-1-yl]-3-phenyl­sydnone

**DOI:** 10.1107/S1600536810033106

**Published:** 2010-08-21

**Authors:** Hoong-Kun Fun, Wan-Sin Loh, Nithinchandra, Balakrishna Kalluraya, Suresh P. Nayak

**Affiliations:** a X-ray Crystallography Unit, School of Physics, Universiti Sains Malaysia, 11800 USM, Penang, Malaysia; b Department of Studies in Chemistry, Mangalore University, Mangalagangotri, Mangalore 574 199, India

## Abstract

In the title compound {systematic name: 4-[4-eth­oxy­carbonyl-5-(3,4-methyl­ene­dioxy­phen­yl)-3-oxocyclo­hex-1-en-1-yl]-3-phenyl-1,2,3-oxadiazol-3-ium-5-olate}, C_24_H_20_N_2_O_7_, the cyclo­hexene and dioxole rings adopt envelope conformations. The sydnone ring and the attached phenyl ring form a dihedral angle of 79.0 (1)°. In the mol­ecular structure, a C—H⋯O hydrogen bond generates an *S*(6) ring and a C—H⋯π inter­action involving the phenyl ring is observed. In the crystal structure, mol­ecules are linked into a ribbon-like structure along the *a* axis by C—H⋯O hydrogen bonds.

## Related literature

For general background and applications of sydnone compounds, see: Rai *et al.* (2008 ▶); Jyothi *et al.* (2008 ▶). For the synthesis of sydnone derivatives, see: Kalluraya *et al.* (2003 ▶). For related structures, see: Goh *et al.* (2010**a* ▶,*b* ▶,c* ▶). For bond-length data, see: Allen *et al.* (1987 ▶). For hydrogen-bond motifs, see: Bernstein *et al.* (1995 ▶). For puckering parameters, see: Cremer & Pople (1975 ▶). For the stability of the temperature controller used in the data collection, see: Cosier & Glazer (1986 ▶). 
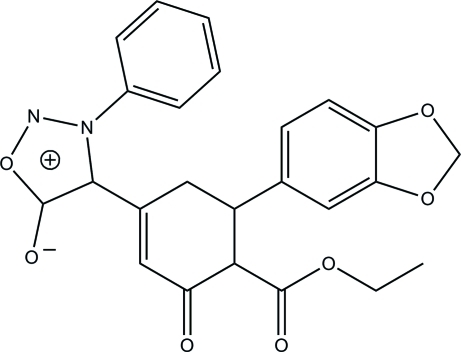


## Experimental

### 

#### Crystal data

C_24_H_20_N_2_O_7_
*M* *_r_* = 448.42
Triclinic, 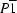

*a* = 8.8026 (2) Å
*b* = 11.5133 (2) Å
*c* = 11.6981 (2) Å
α = 66.860 (1)°
β = 86.545 (1)°
γ = 71.115 (1)°
*V* = 1028.44 (4) Å^3^
*Z* = 2
Mo *K*α radiation
μ = 0.11 mm^−1^
*T* = 100 K
0.37 × 0.13 × 0.06 mm

#### Data collection

Bruker SMART APEXII CCD area-detector diffractometer
Absorption correction: multi-scan (*SADABS*; Bruker, 2009 ▶) *T*_min_ = 0.962, *T*_max_ = 0.994
18550 measured reflections
4703 independent reflections
3710 reflections with *I* > 2σ(*I*)
*R*_int_ = 0.035

#### Refinement

*R*[*F* ^2^ > 2σ(*F* ^2^)] = 0.048
*wR*(*F* ^2^) = 0.119
*S* = 1.03
4703 reflections
299 parameters
H-atom parameters constrained
Δρ_max_ = 0.56 e Å^−3^
Δρ_min_ = −0.34 e Å^−3^

### 

Data collection: *APEX2* (Bruker, 2009 ▶); cell refinement: *SAINT* (Bruker, 2009 ▶); data reduction: *SAINT*; program(s) used to solve structure: *SHELXTL* (Sheldrick, 2008 ▶); program(s) used to refine structure: *SHELXTL*; molecular graphics: *SHELXTL*; software used to prepare material for publication: *SHELXTL* and *PLATON* (Spek, 2009 ▶).

## Supplementary Material

Crystal structure: contains datablocks global, I. DOI: 10.1107/S1600536810033106/ci5156sup1.cif

Structure factors: contains datablocks I. DOI: 10.1107/S1600536810033106/ci5156Isup2.hkl

Additional supplementary materials:  crystallographic information; 3D view; checkCIF report

## Figures and Tables

**Table 1:** Hydrogen-bond geometry (Å, °) *Cg*1 is the centroid of the C1–C6 ring.

*D*—H⋯*A*	*D*—H	H⋯*A*	*D*⋯*A*	*D*—H⋯*A*
C4—H4*A*⋯O4^i^	0.93	2.49	3.304 (3)	146
C5—H5*A*⋯O7^ii^	0.93	2.44	3.258 (2)	146
C14—H14*A*⋯O6	0.93	2.29	2.998 (2)	133
C10—H10*A*⋯*Cg*1	0.97	2.48	3.570 (2)	133

Symmetry codes: (i) 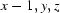; (ii) 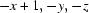.

**Fig. 1.**
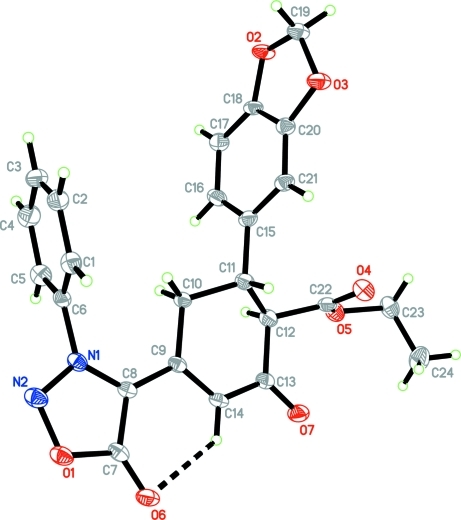
The molecular structure of the title compound, showing 50% probability displacement ellipsoids and the atom-numbering scheme. The dashed line indicates a hydrogen bond.

**Fig. 2.**
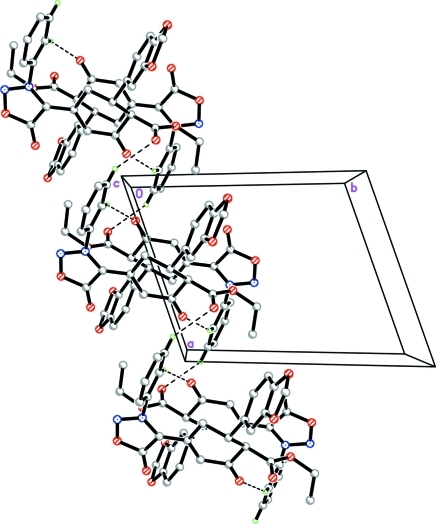
The crystal packing of the title compound showing a hydrogen-bonded ribbon, along the a axis. H atoms not involved in hydrogen bonding (dashed lines) have been omitted for clarity.

## supplementary crystallographic information

### Comment

Sydnones constitute a well defined class of mesoionic compounds that contain the 1,2,3-oxadiazole ring system. The study of sydnones still remains a field of interest because of their electronic structures and also because of the varied types of biological activities displayed by some of them (Rai *et al.*, 2008). Recently sydnone derivatives have been found to exhibit promising antimicrobial properties (Jyothi *et al.*, 2008). The base-catalyzed condensation of 4-acetyl-3-phenyl sydnones with pipernol in aqueous alcoholic medium at 0–50°C gave chalcones. Michael addition of chalcones with ethyl acetoacetate in presence of K_2_CO_3_, followed by Claisen condensation afforded 3-aryl-4-[6-carbethoxy-5-(3,4-methylenedioxyphenyl)cyclohex-2-en-1-one-3yl] phenylsydnone (Kalluraya *et al.*, 2003).

In the title molecule (Fig.1), the cyclohexene ring (C9–C14) adopts an envelope conformation, with the puckering parameters Q = 0.495 (2) Å, Θ = 55.7 (2)°, φ = 126.6 (3)° (Cremer & Pople, 1975). The dioxole ring also adopts an envelope conformation with atom C19 as the flap. The dihedral angle between the sydnone ring and the attached phenyl ring is 79.0 (1)°. The bond lengths (Allen *et al.*, 1987) and angles are comparable to related structures (Goh *et al.*, 2010**a*,*b*,c*). An intramolecular C14—H14A···O6 hydrogen bond (Table 1) generates an *S*(6) ring motif (Fig. 1, Bernstein *et al.*, 1995). An intramolecular C—H···π interaction (Table 1) involving the C1–C6 ring is also observed.

In the crystal packing, intermolecular C4—H4A···O4 and C5—H5A···O7 hydrogen bonds (Table 1) link the molecules into a ribbon-like structure along the *a* axis (Fig. 2).

### Experimental

To a solution of 1-(3-phenylsydnonyl)-3(3,4-methylenedioxyphenyl)-2-propen-1-one (0.01 mol) in dry acetone (50 ml) was added dry potassium carbonate (0.04 mol) and ethyl acetoacetate (0.02 mol) and the mixture was stirred at room temperature overnight and was filtered. The solvent from the filtrate on evaporation gave a solid which was recrystallized from a mixture of ethanol-dioxan. Single crystals suitable for X-ray analysis were obtained from a ethanol solution by slow evaporation.

### Refinement

H atoms were positioned geometrically and refined using a riding model with *U*_iso_(H) = 1.2 or 1.5 *U*_eq_(C) [C–H = 0.93 to 0.97 Å]. A rotating group model was applied to the methyl group.

### Crystal data

**Table:**

C_24_H_20_N_2_O_7_	*Z* = 2
*M**_r_* = 448.42	*F*(000) = 468
Triclinic, *P*1	*D*_x_ = 1.448 Mg m^−^^3^
Hall symbol: -P 1	Mo *K*α radiation, λ = 0.71073 Å
*a* = 8.8026 (2) Å	Cell parameters from 5688 reflections
*b* = 11.5133 (2) Å	θ = 2.2–30.2°
*c* = 11.6981 (2) Å	µ = 0.11 mm^−^^1^
α = 66.860 (1)°	*T* = 100 K
β = 86.545 (1)°	Needle, colourless
γ = 71.115 (1)°	0.37 × 0.13 × 0.06 mm
*V* = 1028.44 (4) Å^3^	

### Data collection

**Table:**

Bruker SMART APEXII CCD area-detector diffractometer	4703 independent reflections
Radiation source: fine-focus sealed tube	3710 reflections with *I* > 2σ(*I*)
graphite	*R*_int_ = 0.035
φ and ω scans	θ_max_ = 27.5°, θ_min_ = 1.9°
Absorption correction: multi-scan (*SADABS*; Bruker, 2009)	*h* = −11→11
*T*_min_ = 0.962, *T*_max_ = 0.994	*k* = −14→14
18550 measured reflections	*l* = −15→15

### Refinement

**Table:**

Refinement on *F*^2^	Primary atom site location: structure-invariant direct methods
Least-squares matrix: full	Secondary atom site location: difference Fourier map
*R*[*F*^2^ > 2σ(*F*^2^)] = 0.048	Hydrogen site location: inferred from neighbouring sites
*wR*(*F*^2^) = 0.119	H-atom parameters constrained
*S* = 1.03	*w* = 1/[σ^2^(*F*_o_^2^) + (0.0499*P*)^2^ + 0.6557*P*] where *P* = (*F*_o_^2^ + 2*F*_c_^2^)/3
4703 reflections	(Δ/σ)_max_ = 0.001
299 parameters	Δρ_max_ = 0.56 e Å^−^^3^
0 restraints	Δρ_min_ = −0.34 e Å^−^^3^

### Special details

**Table:**

Experimental. The crystal was placed in the cold stream of an Oxford Cryosystems Cobra open-flow nitrogen cryostat (Cosier & Glazer, 1986) operating at 100.0 (1) K.
Geometry. All e.s.d.'s (except the e.s.d. in the dihedral angle between two l.s. planes) are estimated using the full covariance matrix. The cell e.s.d.'s are taken into account individually in the estimation of e.s.d.'s in distances, angles and torsion angles; correlations between e.s.d.'s in cell parameters are only used when they are defined by crystal symmetry. An approximate (isotropic) treatment of cell e.s.d.'s is used for estimating e.s.d.'s involving l.s. planes.
Refinement. Refinement of *F*^2^ against ALL reflections. The weighted *R*-factor *wR* and goodness of fit *S* are based on *F*^2^, conventional *R*-factors *R* are based on *F*, with *F* set to zero for negative *F*^2^. The threshold expression of *F*^2^ > σ(*F*^2^) is used only for calculating *R*-factors(gt) *etc*. and is not relevant to the choice of reflections for refinement. *R*-factors based on *F*^2^ are statistically about twice as large as those based on *F*, and *R*- factors based on ALL data will be even larger.

### Fractional atomic coordinates and isotropic or equivalent isotropic displacement parameters (Å^2^)

**Table:**

	*x*	*y*	*z*	*U*_iso_*/*U*_eq_	
O1	0.54796 (15)	−0.47982 (12)	0.24568 (11)	0.0225 (3)	
O2	0.09931 (15)	0.43102 (12)	0.42387 (11)	0.0228 (3)	
O3	0.34786 (15)	0.29504 (13)	0.53026 (11)	0.0244 (3)	
O4	0.73361 (16)	0.19987 (13)	0.20782 (12)	0.0263 (3)	
O5	0.63381 (15)	0.31720 (12)	0.00796 (11)	0.0234 (3)	
O6	0.73888 (15)	−0.39479 (12)	0.14030 (12)	0.0249 (3)	
O7	0.79429 (16)	0.03513 (13)	0.01666 (12)	0.0271 (3)	
N1	0.39657 (17)	−0.31654 (14)	0.28604 (13)	0.0175 (3)	
N2	0.41071 (19)	−0.43911 (14)	0.30166 (14)	0.0227 (3)	
C1	0.2822 (2)	−0.29087 (18)	0.47181 (16)	0.0219 (4)	
H1A	0.3768	−0.3542	0.5175	0.026*	
C2	0.1577 (2)	−0.23095 (19)	0.52984 (17)	0.0255 (4)	
H2A	0.1680	−0.2536	0.6152	0.031*	
C3	0.0177 (2)	−0.13688 (19)	0.45938 (19)	0.0279 (4)	
H3A	−0.0658	−0.0963	0.4980	0.033*	
C4	0.0010 (2)	−0.10279 (19)	0.33208 (18)	0.0277 (4)	
H4A	−0.0935	−0.0395	0.2861	0.033*	
C5	0.1236 (2)	−0.16201 (18)	0.27268 (16)	0.0231 (4)	
H5A	0.1129	−0.1404	0.1875	0.028*	
C6	0.2628 (2)	−0.25450 (16)	0.34455 (15)	0.0181 (3)	
C7	0.6164 (2)	−0.37744 (16)	0.19344 (15)	0.0186 (4)	
C8	0.5132 (2)	−0.26951 (16)	0.22242 (15)	0.0165 (3)	
C9	0.5348 (2)	−0.14190 (16)	0.19358 (14)	0.0155 (3)	
C10	0.4237 (2)	−0.03949 (16)	0.23803 (15)	0.0161 (3)	
H10A	0.3924	−0.0853	0.3202	0.019*	
H10B	0.3269	0.0099	0.1819	0.019*	
C11	0.5027 (2)	0.05872 (17)	0.24418 (16)	0.0196 (4)	
H11A	0.5949	0.0075	0.3064	0.023*	
C12	0.5672 (2)	0.12241 (17)	0.11849 (16)	0.0209 (4)	
H12A	0.4763	0.1728	0.0550	0.025*	
C13	0.6849 (2)	0.01333 (18)	0.08194 (16)	0.0200 (4)	
C14	0.6583 (2)	−0.11497 (17)	0.12458 (15)	0.0177 (3)	
H14A	0.7294	−0.1819	0.1035	0.021*	
C15	0.3878 (2)	0.16253 (17)	0.28630 (16)	0.0193 (4)	
C16	0.2380 (2)	0.24512 (18)	0.22215 (16)	0.0225 (4)	
H16A	0.2090	0.2370	0.1512	0.027*	
C17	0.1295 (2)	0.34015 (17)	0.26119 (16)	0.0212 (4)	
H17A	0.0298	0.3947	0.2183	0.025*	
C18	0.1795 (2)	0.34732 (16)	0.36599 (15)	0.0180 (4)	
C19	0.1948 (2)	0.38560 (18)	0.53747 (17)	0.0232 (4)	
H19A	0.1418	0.3407	0.6080	0.028*	
H19B	0.2093	0.4608	0.5482	0.028*	
C20	0.3280 (2)	0.26646 (17)	0.42936 (16)	0.0190 (4)	
C21	0.4350 (2)	0.17364 (17)	0.39255 (16)	0.0193 (4)	
H21A	0.5346	0.1204	0.4362	0.023*	
C22	0.6534 (2)	0.21628 (17)	0.11888 (16)	0.0187 (4)	
C23	0.7312 (2)	0.40341 (18)	−0.00653 (18)	0.0267 (4)	
H23A	0.7349	0.4177	0.0695	0.032*	
H23B	0.6825	0.4896	−0.0740	0.032*	
C24	0.8997 (2)	0.3393 (2)	−0.0344 (2)	0.0348 (5)	
H24A	0.9649	0.3929	−0.0374	0.052*	
H24B	0.8965	0.3325	−0.1134	0.052*	
H24C	0.9450	0.2516	0.0298	0.052*	

### Atomic displacement parameters (Å^2^)

**Table:**

	*U*^11^	*U*^22^	*U*^33^	*U*^12^	*U*^13^	*U*^23^
O1	0.0288 (7)	0.0144 (6)	0.0231 (6)	−0.0049 (5)	0.0041 (5)	−0.0082 (5)
O2	0.0216 (6)	0.0217 (6)	0.0233 (6)	0.0021 (5)	0.0006 (5)	−0.0142 (5)
O3	0.0209 (7)	0.0281 (7)	0.0225 (6)	0.0006 (5)	−0.0002 (5)	−0.0150 (5)
O4	0.0303 (7)	0.0250 (7)	0.0251 (7)	−0.0094 (6)	0.0006 (5)	−0.0109 (5)
O5	0.0281 (7)	0.0187 (6)	0.0231 (6)	−0.0081 (5)	0.0003 (5)	−0.0076 (5)
O6	0.0263 (7)	0.0211 (7)	0.0299 (7)	−0.0048 (5)	0.0064 (5)	−0.0158 (5)
O7	0.0295 (7)	0.0278 (7)	0.0315 (7)	−0.0141 (6)	0.0156 (6)	−0.0175 (6)
N1	0.0233 (8)	0.0134 (7)	0.0153 (7)	−0.0058 (6)	0.0011 (5)	−0.0052 (5)
N2	0.0304 (9)	0.0148 (7)	0.0217 (7)	−0.0066 (6)	0.0047 (6)	−0.0069 (6)
C1	0.0248 (9)	0.0215 (9)	0.0191 (8)	−0.0096 (7)	0.0013 (7)	−0.0061 (7)
C2	0.0312 (10)	0.0313 (10)	0.0212 (9)	−0.0166 (9)	0.0083 (7)	−0.0134 (8)
C3	0.0280 (10)	0.0265 (10)	0.0360 (11)	−0.0117 (8)	0.0134 (8)	−0.0185 (8)
C4	0.0237 (10)	0.0201 (9)	0.0345 (10)	−0.0037 (8)	0.0019 (8)	−0.0087 (8)
C5	0.0281 (10)	0.0198 (9)	0.0193 (8)	−0.0082 (8)	0.0008 (7)	−0.0053 (7)
C6	0.0221 (9)	0.0152 (8)	0.0200 (8)	−0.0087 (7)	0.0053 (7)	−0.0085 (6)
C7	0.0246 (9)	0.0138 (8)	0.0166 (8)	−0.0040 (7)	−0.0021 (7)	−0.0062 (6)
C8	0.0190 (8)	0.0156 (8)	0.0150 (8)	−0.0039 (7)	0.0010 (6)	−0.0074 (6)
C9	0.0185 (8)	0.0149 (8)	0.0125 (7)	−0.0032 (6)	−0.0018 (6)	−0.0062 (6)
C10	0.0179 (8)	0.0141 (8)	0.0167 (8)	−0.0044 (6)	0.0029 (6)	−0.0073 (6)
C11	0.0216 (9)	0.0179 (8)	0.0210 (8)	−0.0062 (7)	0.0035 (7)	−0.0099 (7)
C12	0.0238 (9)	0.0194 (9)	0.0214 (9)	−0.0073 (7)	0.0028 (7)	−0.0101 (7)
C13	0.0225 (9)	0.0232 (9)	0.0188 (8)	−0.0086 (7)	0.0044 (7)	−0.0122 (7)
C14	0.0200 (8)	0.0175 (8)	0.0172 (8)	−0.0032 (7)	0.0023 (6)	−0.0109 (6)
C15	0.0199 (9)	0.0152 (8)	0.0237 (9)	−0.0068 (7)	0.0083 (7)	−0.0086 (7)
C16	0.0287 (10)	0.0235 (9)	0.0203 (8)	−0.0105 (8)	0.0051 (7)	−0.0127 (7)
C17	0.0220 (9)	0.0183 (9)	0.0211 (9)	−0.0046 (7)	0.0013 (7)	−0.0070 (7)
C18	0.0199 (9)	0.0129 (8)	0.0207 (8)	−0.0036 (7)	0.0070 (7)	−0.0081 (6)
C19	0.0234 (9)	0.0227 (9)	0.0235 (9)	−0.0024 (7)	0.0021 (7)	−0.0132 (7)
C20	0.0212 (9)	0.0164 (8)	0.0195 (8)	−0.0063 (7)	0.0043 (7)	−0.0075 (6)
C21	0.0179 (8)	0.0151 (8)	0.0225 (9)	−0.0033 (7)	0.0041 (7)	−0.0071 (7)
C22	0.0187 (8)	0.0175 (8)	0.0210 (8)	−0.0038 (7)	0.0051 (7)	−0.0108 (7)
C23	0.0346 (11)	0.0187 (9)	0.0287 (10)	−0.0136 (8)	0.0014 (8)	−0.0071 (7)
C24	0.0315 (11)	0.0345 (12)	0.0395 (12)	−0.0176 (9)	0.0060 (9)	−0.0107 (9)

### Geometric parameters (Å, °)

**Table:**

O1—N2	1.3739 (19)	C10—C11	1.532 (2)
O1—C7	1.405 (2)	C10—H10A	0.97
O2—C18	1.379 (2)	C10—H10B	0.97
O2—C19	1.432 (2)	C11—C15	1.521 (2)
O3—C20	1.378 (2)	C11—C12	1.531 (2)
O3—C19	1.434 (2)	C11—H11A	0.98
O4—C22	1.210 (2)	C12—C22	1.509 (2)
O5—C22	1.333 (2)	C12—C13	1.536 (2)
O5—C23	1.466 (2)	C12—H12A	0.98
O6—C7	1.211 (2)	C13—C14	1.455 (2)
O7—C13	1.221 (2)	C14—H14A	0.93
N1—N2	1.314 (2)	C15—C16	1.397 (3)
N1—C8	1.361 (2)	C15—C21	1.399 (2)
N1—C6	1.452 (2)	C16—C17	1.408 (2)
C1—C6	1.384 (2)	C16—H16A	0.93
C1—C2	1.388 (3)	C17—C18	1.368 (2)
C1—H1A	0.93	C17—H17A	0.93
C2—C3	1.389 (3)	C18—C20	1.383 (2)
C2—H2A	0.93	C19—H19A	0.97
C3—C4	1.387 (3)	C19—H19B	0.97
C3—H3A	0.93	C20—C21	1.370 (2)
C4—C5	1.385 (3)	C21—H21A	0.93
C4—H4A	0.93	C23—C24	1.508 (3)
C5—C6	1.385 (2)	C23—H23A	0.97
C5—H5A	0.9300	C23—H23B	0.97
C7—C8	1.431 (2)	C24—H24A	0.96
C8—C9	1.446 (2)	C24—H24B	0.96
C9—C14	1.358 (2)	C24—H24C	0.96
C9—C10	1.514 (2)		
			
N2—O1—C7	110.82 (12)	C11—C12—C13	110.07 (14)
C18—O2—C19	105.46 (13)	C22—C12—H12A	108.5
C20—O3—C19	105.33 (13)	C11—C12—H12A	108.5
C22—O5—C23	115.58 (14)	C13—C12—H12A	108.5
N2—N1—C8	115.22 (14)	O7—C13—C14	121.36 (16)
N2—N1—C6	114.75 (14)	O7—C13—C12	120.91 (16)
C8—N1—C6	129.96 (14)	C14—C13—C12	117.71 (15)
N1—N2—O1	104.74 (13)	C9—C14—C13	123.30 (16)
C6—C1—C2	118.67 (17)	C9—C14—H14A	118.4
C6—C1—H1A	120.7	C13—C14—H14A	118.4
C2—C1—H1A	120.7	C16—C15—C21	119.99 (16)
C1—C2—C3	119.48 (17)	C16—C15—C11	121.71 (16)
C1—C2—H2A	120.3	C21—C15—C11	118.30 (15)
C3—C2—H2A	120.3	C15—C16—C17	122.31 (16)
C4—C3—C2	120.67 (18)	C15—C16—H16A	118.8
C4—C3—H3A	119.7	C17—C16—H16A	118.8
C2—C3—H3A	119.7	C18—C17—C16	115.96 (16)
C5—C4—C3	120.66 (18)	C18—C17—H17A	122.0
C5—C4—H4A	119.7	C16—C17—H17A	122.0
C3—C4—H4A	119.7	C17—C18—O2	128.32 (16)
C6—C5—C4	117.67 (17)	C17—C18—C20	121.97 (16)
C6—C5—H5A	121.2	O2—C18—C20	109.71 (15)
C4—C5—H5A	121.2	O2—C19—O3	107.89 (13)
C1—C6—C5	122.84 (16)	O2—C19—H19A	110.1
C1—C6—N1	117.38 (15)	O3—C19—H19A	110.1
C5—C6—N1	119.77 (15)	O2—C19—H19B	110.1
O6—C7—O1	120.52 (15)	O3—C19—H19B	110.1
O6—C7—C8	134.77 (16)	H19A—C19—H19B	108.4
O1—C7—C8	104.70 (14)	C21—C20—O3	127.31 (16)
N1—C8—C7	104.51 (14)	C21—C20—C18	122.79 (16)
N1—C8—C9	128.77 (15)	O3—C20—C18	109.90 (15)
C7—C8—C9	126.68 (15)	C20—C21—C15	116.97 (16)
C14—C9—C8	118.32 (15)	C20—C21—H21A	121.5
C14—C9—C10	120.05 (15)	C15—C21—H21A	121.5
C8—C9—C10	121.62 (14)	O4—C22—O5	124.34 (16)
C9—C10—C11	112.31 (14)	O4—C22—C12	124.18 (16)
C9—C10—H10A	109.1	O5—C22—C12	111.46 (14)
C11—C10—H10A	109.1	O5—C23—C24	109.97 (15)
C9—C10—H10B	109.1	O5—C23—H23A	109.7
C11—C10—H10B	109.1	C24—C23—H23A	109.7
H10A—C10—H10B	107.9	O5—C23—H23B	109.7
C15—C11—C12	112.34 (14)	C24—C23—H23B	109.7
C15—C11—C10	111.42 (14)	H23A—C23—H23B	108.2
C12—C11—C10	109.94 (14)	C23—C24—H24A	109.5
C15—C11—H11A	107.6	C23—C24—H24B	109.5
C12—C11—H11A	107.6	H24A—C24—H24B	109.5
C10—C11—H11A	107.6	C23—C24—H24C	109.5
C22—C12—C11	113.06 (14)	H24A—C24—H24C	109.5
C22—C12—C13	108.19 (14)	H24B—C24—H24C	109.5
			
C8—N1—N2—O1	0.58 (18)	C11—C12—C13—O7	−151.39 (16)
C6—N1—N2—O1	−176.69 (13)	C22—C12—C13—C14	154.41 (15)
C7—O1—N2—N1	−0.90 (17)	C11—C12—C13—C14	30.4 (2)
C6—C1—C2—C3	0.1 (3)	C8—C9—C14—C13	175.29 (15)
C1—C2—C3—C4	0.2 (3)	C10—C9—C14—C13	−4.6 (2)
C2—C3—C4—C5	0.1 (3)	O7—C13—C14—C9	−177.63 (16)
C3—C4—C5—C6	−0.7 (3)	C12—C13—C14—C9	0.5 (2)
C2—C1—C6—C5	−0.8 (3)	C12—C11—C15—C16	66.7 (2)
C2—C1—C6—N1	178.54 (15)	C10—C11—C15—C16	−57.2 (2)
C4—C5—C6—C1	1.1 (3)	C12—C11—C15—C21	−113.64 (17)
C4—C5—C6—N1	−178.24 (15)	C10—C11—C15—C21	122.49 (16)
N2—N1—C6—C1	78.33 (19)	C21—C15—C16—C17	−0.7 (3)
C8—N1—C6—C1	−98.4 (2)	C11—C15—C16—C17	178.99 (16)
N2—N1—C6—C5	−102.29 (18)	C15—C16—C17—C18	0.2 (3)
C8—N1—C6—C5	80.9 (2)	C16—C17—C18—O2	179.39 (16)
N2—O1—C7—O6	179.69 (15)	C16—C17—C18—C20	0.3 (3)
N2—O1—C7—C8	0.88 (17)	C19—O2—C18—C17	172.59 (18)
N2—N1—C8—C7	−0.05 (19)	C19—O2—C18—C20	−8.19 (18)
C6—N1—C8—C7	176.72 (15)	C18—O2—C19—O3	12.89 (18)
N2—N1—C8—C9	−177.79 (16)	C20—O3—C19—O2	−12.72 (18)
C6—N1—C8—C9	−1.0 (3)	C19—O3—C20—C21	−172.51 (17)
O6—C7—C8—N1	−179.06 (19)	C19—O3—C20—C18	7.75 (18)
O1—C7—C8—N1	−0.50 (17)	C17—C18—C20—C21	−0.2 (3)
O6—C7—C8—C9	−1.3 (3)	O2—C18—C20—C21	−179.48 (15)
O1—C7—C8—C9	177.31 (15)	C17—C18—C20—O3	179.55 (15)
N1—C8—C9—C14	−177.80 (16)	O2—C18—C20—O3	0.27 (19)
C7—C8—C9—C14	4.9 (3)	O3—C20—C21—C15	180.00 (16)
N1—C8—C9—C10	2.0 (3)	C18—C20—C21—C15	−0.3 (3)
C7—C8—C9—C10	−175.23 (15)	C16—C15—C21—C20	0.7 (2)
C14—C9—C10—C11	−23.1 (2)	C11—C15—C21—C20	−178.98 (15)
C8—C9—C10—C11	157.03 (15)	C23—O5—C22—O4	6.4 (2)
C9—C10—C11—C15	178.75 (14)	C23—O5—C22—C12	−171.70 (14)
C9—C10—C11—C12	53.54 (18)	C11—C12—C22—O4	34.7 (2)
C15—C11—C12—C22	57.8 (2)	C13—C12—C22—O4	−87.5 (2)
C10—C11—C12—C22	−177.53 (14)	C11—C12—C22—O5	−147.17 (15)
C15—C11—C12—C13	178.89 (14)	C13—C12—C22—O5	90.67 (17)
C10—C11—C12—C13	−56.42 (18)	C22—O5—C23—C24	80.80 (19)
C22—C12—C13—O7	−27.4 (2)		

### Hydrogen-bond geometry (Å, °)

**Table:**

Cg1 is the centroid of the C1–C6 ring.

**Table:**

*D*—H···*A*	*D*—H	H···*A*	*D*···*A*	*D*—H···*A*
C4—H4A···O4^i^	0.93	2.49	3.304 (3)	146
C5—H5A···O7^ii^	0.93	2.44	3.258 (2)	146
C14—H14A···O6	0.93	2.29	2.998 (2)	133
C10—H10A···Cg1	0.97	2.48	3.570 (2)	133

Symmetry codes: (i) *x*−1, *y*, *z*; (ii) −*x*+1, −*y*, −*z*.
